# Emergence of ITD tuning in the MSO with a realistic periphery model

**DOI:** 10.1186/1471-2202-16-S1-P20

**Published:** 2015-12-18

**Authors:** P Yger, V Benichoux, M Stimberg, R Brette

**Affiliations:** 1 Institut d'Etudes de la Cognition, Ecole Normale Supérieure, Paris, France; 2 Sorbonne Universités, UPMC Univ. Paris 06, UMR S 968, Institut de la Vision, Paris, F-75012, France; 3 INSERM, U968, Paris, F-75012, France; 4 CNRS, UMR 7210, Paris, F-75012, France

## 

To localize sounds in the environment, animals mostly rely on spectro-temporal cues originating from the physical disparities of the sound waveforms impacting the two ears. Among those, the Interaural Time Difference (ITD) has been shown to be crucial in mammals for locating low-frequency sounds, and is known to be processed by neurons in a particular structure, the Medial Superior Olive (MSO). While it is classically considered that the emergence of ITD selectivity in a neuron of the MSO is simply due to differences in the axonal delays originating from the two ears and impinging those neurons (the so called "delay-line" model [1]), experimental evidence shows that the best delay (the ITD at which the neuron's firing rate is maximum) is also dependent on the frequency of the sound [2].

To investigate more on this challenging experimental observation, we developed a realistic periphery model to mimic cochlear inputs from auditory nerves fibers onto the MSO. Using known plasticity rules such as Spike Timing Dependent Plasticity to structure the wiring from those connections onto the MSO, we extend the work that has already been performed [3], and study the emergence of binaural tuning in the MSO in a realistic scenario. The system is trained with binaural sounds such as white noise, and then, as in most experimental papers, we tested the ITD selectivity of cells in the MSO by presenting pure tones at various frequencies.

Finally, we discuss, from a coding point of view the potential implications raised by the frequency dependence of the best delay. As pointed out by recent work [4], with such a frequency-dependent best delay, neurons in the MSO should be seen as coding for a particular position in space, rather than for just a fixed delay difference.
